# Improving truffle mycelium flavour through strain selection targeting volatiles of the Ehrlich pathway

**DOI:** 10.1038/s41598-018-27620-w

**Published:** 2018-06-18

**Authors:** Maryam Vahdatzadeh, Richard Splivallo

**Affiliations:** 1 Goethe University Frankfurt, Institute for Molecular Biosciences, 60438 Frankfurt, Germany; 2 Integrative Fungal Research Cluster (IPF), 60325 Frankfurt, Germany

## Abstract

Truffles (*Tuber* spp.) are the fruiting bodies of symbiotic fungi, which are prized food delicacies. The marked aroma variability observed among truffles of the same species has been attributed to a series of factors that are still debated. This is because factors (i.e. genetics, maturation, geographical location and the microbial community colonizing truffles) often co-vary in truffle orchards. Here, we removed the co-variance effect by investigating truffle flavour in axenic cultures of nine strains of the white truffle *Tuber borchii*. This allowed us to investigate the influence of genetics on truffle aroma. Specifically, we quantified aroma variability and explored whether strain selection could be used to improve human-sensed truffle flavour. Our results illustrate that aroma variability among strains is predominantly linked to amino acid catabolism through the Ehrlich pathway, as confirmed by ^13^C labelling experiments. We furthermore exemplified through sensory analysis that the human nose is able to distinguish among strains and that sulfur volatiles derived from the catabolism of methionine have the strongest influence on aroma characteristics. Overall, our results demonstrate that genetics influences truffle aroma much more deeply than previously thought and illustrate the usefulness of strain selection for improving truffle flavour.

## Introduction

Truffles (*Tuber* spp.), fruiting bodies of symbiotic fungi which develop underground, are well known for their enticing and captivating aromas^1^. They are edible fungal-organ bearing spores, which result from sexual reproduction^2–4^. Indeed, truffles are heterothallic organisms, meaning that they need two mating types (maternal and paternal) for reproduction. Mating occurs once the maternal mating type that colonizes plant roots in a symbiotic organ known as ectomycorrhizas encounters an individual of opposite (paternal) mating type. This results in the formation of a haploid fruiting body containing spores of both mating types^4^. Molecular based data suggest the existence of about 180 truffle species in various regions of the world^5^, of which about 30 are commercially traded. Prices range from a few hundred Euros per kg for the cheapest truffle species up to thousands of Euros for the most expensive ones such as the white Piedmont truffle *Tuber magnatum* or the Périgord black truffle *Tuber melanosporum*.

Truffle fruiting bodies are considered a food delicacy, mostly due to their unique aromas^1^. Volatiles responsible for their distinctive smells are a blend of alcohols, ketones, aldehydes, aromatic and sulfur compounds. As with any other food products^6^, only a small fraction of all volatiles emitted by truffles (the so-called odorants) are responsible for the smell perceived by humans^7–9^. In terms of composition, certain odorants are common to many truffle species while others are species specific or limited to a few species only. For example, 2-methylbutanal, 3-methylbutanal, 2- methylbutan-1-ol, 3-methylbutanol and oct-1-en-3-ol are common to most truffle species^10^ while 2,4-dithiapentane and 3-methyl-4,5-(2 H)thiophene have been exclusively described in fruiting bodies of the white species *T*. *magnatum*, and *Tuber borchii*, respectively^7,11,12^. The volatile composition can vary throughout the various stages of a truffle’s life cycle. This is for instance illustrated by 3-methyl-4,5-(2 H)thiophene that seems to be exclusively emitted during the sexual stage (fruiting bodies) of *T*. *borchii*. This compound is indeed not detectable from axenic mycelial cultures, even when they are re-inoculated with bacteria that produce this volatile in fruiting bodies^12^. Overall, the volatile profile of axenic cultures of truffle mycelium tends to be less complex in terms of the number of compounds than one of truffle fruiting bodies^13,14^. This disparity is possibly caused by differences in developmental stages or the lack of interacting microbes as highlighted hereafter^15^.

Truffle fruiting bodies are heavily colonized by bacteria and to a lesser extent by yeasts, filamentous fungi and viruses^15^. The origin of many truffle odorants is hence unclear, as they might be synthesized by the truffle itself or by its microbiome. The contribution of microbes to truffle aroma has indeed only been demonstrated in a single case, which links bacteria inhabiting the fruiting body of *T*. *borchii* to the production of thiophene derivatives (i.e. 3-methyl-4,5-(2 H)thiophene)^7,12^. Additionally, indirect evidence suggests that bacteria might be exclusively responsible for the emission of 2,4-dithiapentane in *T*. *magnatum* as well^15^. By contrast, the origin of odorants that are more common among truffles (i.e. dimethyl sulfide, 2- methylbutan-1-ol and 3-methylbutan-1-ol and their aldehydes derivatives, just to cite a few examples) remains elusive since, theoretically, they may be synthesized by both truffles and their microbiomes^15^.

Uncertainties currently exist not only about the origins of truffle odorants, but also about their precursors. Indeed, the identity of aroma precursors in truffles is to a large extent based on speculation and indirect evidence. Following the sequencing of the genome of the black truffle *T*. *melanosporum*^16^, it has been suggested that numerous truffle odorants were produced from amino acid catabolism through the Ehrlich pathway. In this pathway, an amino acid is first deaminated into an α-keto acid, followed by a decarboxylation into an aldehyde and either a reduction or oxidation into an alcohol or acid, respectively^10,16,17^. In this way, leucine, isoleucine, phenylalanine and methionine are respectively transformed into 3-methylbutanal, 2-methylbutanal, 2-phenylacetaldehyde and 3-methylsulfanylpropanal and their corresponding alcohols and acids^17^. Supplying axenic cultures of truffle mycelium with leucine, isoleucine, phenylalanine and methionine was shown to induce numerous volatiles of the Ehrlich pathway^18–20^, suggesting that these amino acids were either the direct precursors of those volatiles or indirectly induced them. Nevertheless, demonstrating the existence of the Ehrlich pathway beyond a reasonable doubt shall ultimately require feeding experiments with isotopically-labelled amino acids, which has not been performed to date.

Differences in the aroma profiles of truffles do not only exist among species and/or developmental stages as highlighted earlier, but also within truffles of the same species. Indeed, a major variability in the concentration of four and eight carbon-containing volatiles (i.e. oct-1-en-3-ol and 2-butanone) has been documented for *Tuber aestivum* fruiting bodies collected a few centimeters apart in the same truffle orchards^21,22^. This aroma variability has been linked to genetic differences^21,22^. Considering that strain selection has been successfully performed in microbes to improve the final flavour of fermented food products (i.e. cheese and wine) or to eliminate off-flavour compounds^23–26^, a similar approach might improve the characteristics of truffle flavour produced through axenic truffle mycelium cultures.

**Figure 1:**
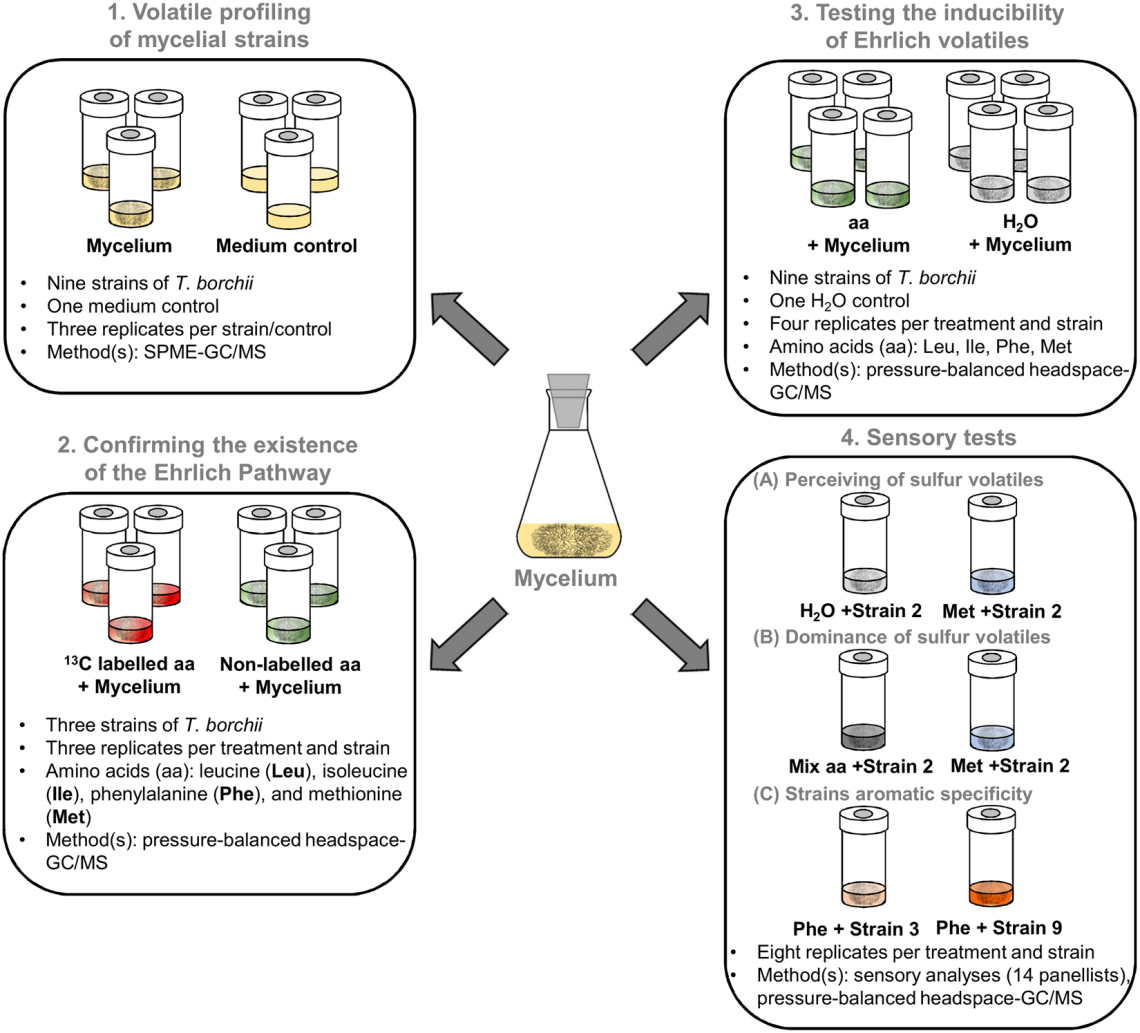
Experimental design. This figure highlights the number of replicates used for each experiment and the techniques employed.

The main aim of this study was to assess whether strain selection could be used for improving human-sensed truffle flavour produced through mycelial fermentation. To answer this, nine strains of the white truffle *T*. *borchii* were tested in various feeding experiments and sensory tests (see Fig. 1 for the experimental design). This species, endemic to Europe and introduced in New Zealand^5^ was chosen because of the good growth of its mycelium compared to other truffle species^27^. Specifically, in the first set of experiments, the extent of aroma variability among strains was assessed and volatiles responsible for this variability were identified. Subsequently, the existence of the Ehrlich pathway was tested with isotopically labelled amino acids and finally, sensory tests were performed to assess whether the human nose was capable of differentiating among strains.

## Results

### *T*. *borchii* mycelia vary in their volatile profiles

**Figure 2:**
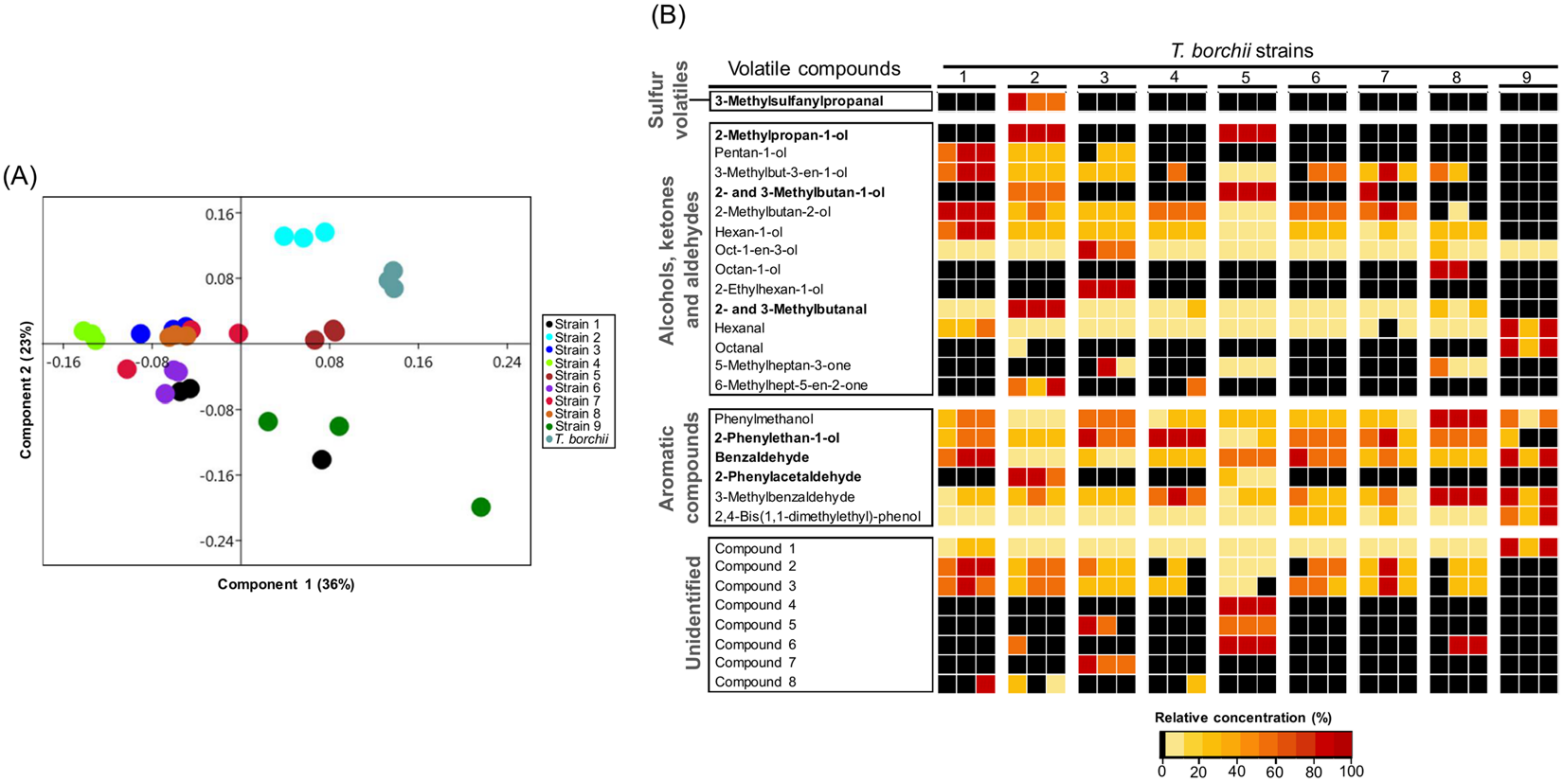
Volatile profiles of nine *T*. *borchii* mycelial strains. (**A**) PCA (based on Supplementary Table S1) illustrates the variability in volatile profile for nine *T*. *borchii* mycelial strains and one fruiting body (n = 3 replicates per sample). (**B**) The heatmap highlights volatile compounds which concentrations significantly differed among strains (n = 3 replicates per strain, p < 0.05, Kruskal-Wallis test with α = 0.05). Volatile compounds derived from the Ehrlich pathway are highlighted in bold.

The aim of this first experiment was to assess the variability in aroma profiles of mycelial cultures of *T*. *borchii*. For this purpose, the aroma profiles of nine strains (axenic cultures grown in malt extract) and one fruiting body (included here for completeness) of *T*. *borchii* (Table 1) were analysed by solid-phase microextraction gas chromatography-mass spectroscopy (SPME-GC/MS). Volatile profiles, generated for three independent replicates of all strains/fruiting body, were processed for peak realignment with the Tagfinder software^28^. This resulted in a data matrix of specific TAGs (volatiles) in each sample (Supplementary Table S1 illustrates the raw data of TAGs normalized to the total ion current (TIC)). Principle Component Analysis (PCA) applied to the volatile profile in Supplementary Table S1 could explain 59% of the data variability as seen in Fig. 2A, highlighting differences among strains.

**Table 1:** *T*. *borchii* strains and their origins.

Mycelium strain or fruiting body	Accession number (GenBank - NCBI)	Accession number reported in	Origin
Strain 1	DQ679802	Bonuso *et al*.^53^	Emilia-Romagna, Italy
Strain 2	FJ554505	Bonuso *et al*.^53^	Emilia-Romagna, Italy
Strain 3	FJ554476	Bonuso *et al*.^53^	Emilia-Romagna, Italy
Strain 4	MF686459	Current work	Piedmont, Italy
Strain 5	KP244305	Splivallo *et al*.^7^	Piedmont, Italy
Strain 6	KF414978	Splivallo *et al*.^12^	Piedmont, Italy
Strain 7	KP244306	Splivallo *et al*.^7^	Piedmont, Italy
Strain 8	KP244307	Splivallo *et al*.^7^	Piedmont, Italy
Strain 9	MF686460	Current work	Canterbury, New Zealand
Fruiting body*	—	—	Piedmont, Italy

*Identified based on spores’ morphology.

TAGs which significantly differed in concentrations among strains were identified using the non-parametric Kruskal-Wallis statistical test performed in R^29^. Those TAGs could be assigned to 29 compounds, which are represented in a heat map in Fig. 2B. These include one sulfur-containing volatile, alkenes, alcohols, aldehydes, ketones, aromatic compounds and eight unidentified volatiles. Both qualitative and quantitative differences were detected among strains. Interestingly, one-third of the volatiles that varied in concentrations among strains might be products of the Ehrlich pathway. Common volatiles produced by most *T*. *borchii* strains included 2- and 3-methylbutanal, benzaldehyde, 2-phenylethan-1-ol whereas other volatiles such as 2-phenylacetaldehyde, 2- and 3-methylbutan-1-ol and 2-methylpropan-1-ol were specific to two or three strains and 3-methylsulfanylpropanal was detected from a single strain (strain 2) only (Fig. 2B).

Our data demonstrated that the largest part of aroma variability among strains was due to quantitative differences in volatiles possibly derived from amino acids catabolism (Ehrlich pathway).

### Confirming the existence of the Ehrlich Pathway in *T*. *borchii* through feeding mycelia with ^13^C labelled amino acids

**Figure 3:**
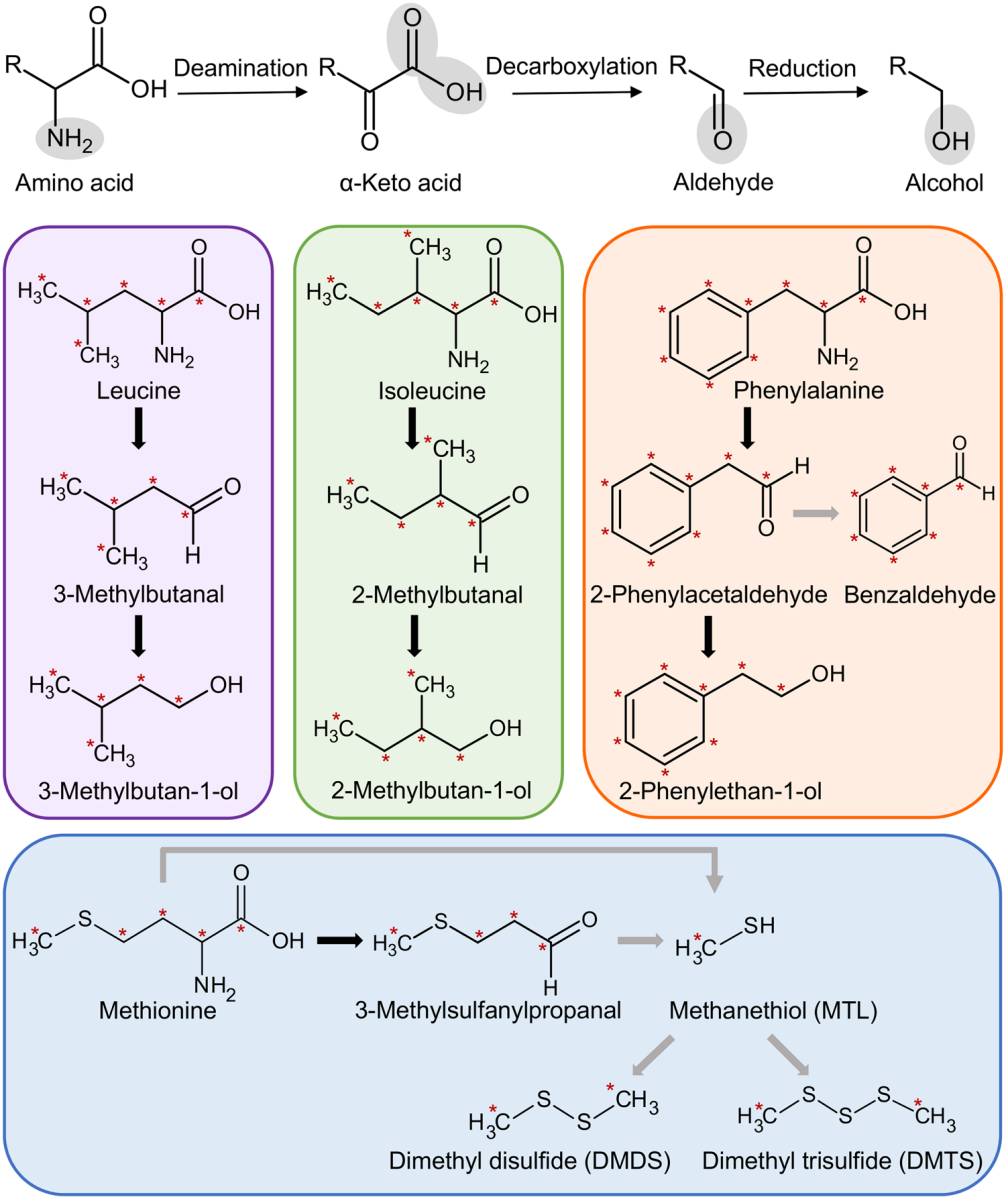
Amino acids catabolism in *Tuber borchii*. The scheme on top illustrates the three steps of the Ehrlich pathway. Specific structures of amino acids and volatile compounds detected in our experiments (with the exception of MTL which was not detected but is shown here for completeness) are shown below with ^13^C label incorporation. Labelling experiments with three mycelial strains (strains 2, 3 and 5) gave comparable results. Refer to Supplementary Fig. S1 for details.

Considering the important variability in volatiles possibly derived from amino acid catabolism, we tested the existence of the Ehrlich pathway in truffles. With this aim in mind, three mycelial strains (strains 2, 3 and 5) were supplied with four amino acids separately, namely leucine, isoleucine, phenylalanine, and methionine (unlabelled and ^13^C labelled) and their aromas were profiled by pressure-balanced headspace-GC/MS. Pressure-balanced headspace extraction and subsequent trapping on a charcoal cartridge was chosen in favour of SPME for its improved reproducibility. In all strains, leucine induced 3-methylbutanal and 3-methylbutan-1-ol; isoleucine: 2-methylbutanal and 2-methylbutan-1-ol; phenylalanine, 2-phenylacetaldehyde, 2-phenylethan-1-ol, and benzaldehyde; methionine, 3-methylsulfanylpropanal, dimethyl disulfide (DMDS), dimethyl trisulfide (DMTS) (Fig. 3). Comparison of the mass spectra of all three mycelial strains supplemented with ^13^C labelled and unlabelled amino acids gave comparable results for all strains and showed that all the labelled carbon atoms were fully incorporated in target volatiles (Fig. 3 and Supplementary Fig. S1), hence confirming the existence of the Ehrlich pathway in *T*. *borchii*.

### Testing the induction of Ehrlich volatiles in *T*. *borchii*

**Figure 4:**
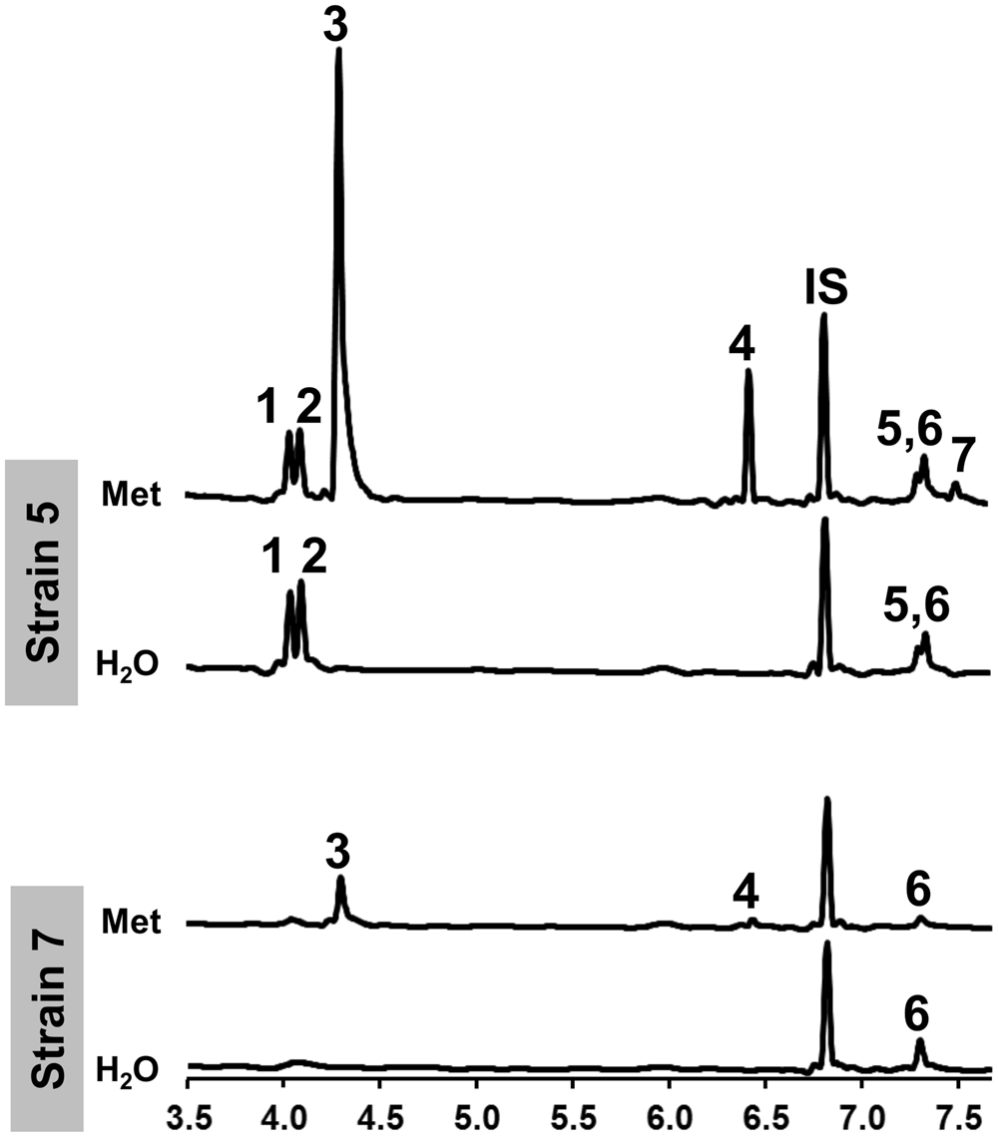
Sulfur volatile compounds induced by methionine in *T*. *borchii*. Chromatograms are shown for two strains supplemented with Met (20 mM) or water (control). The y-axis is comparable for all chromatograms. Note the stronger S-volatile induction of strain 5 compared to strain 7. Volatile compounds: **(1)** 3-methylbutan-1-ol; **(2)** 2-methylbutan-1-ol; **(3)** DMDS; **(4)** 3-methylsulfanylpropanal; **(5)** benzaldehyde; **(6)** unidentified; **(7)** DMTS and IS (internal standard).

**Figure 5:**
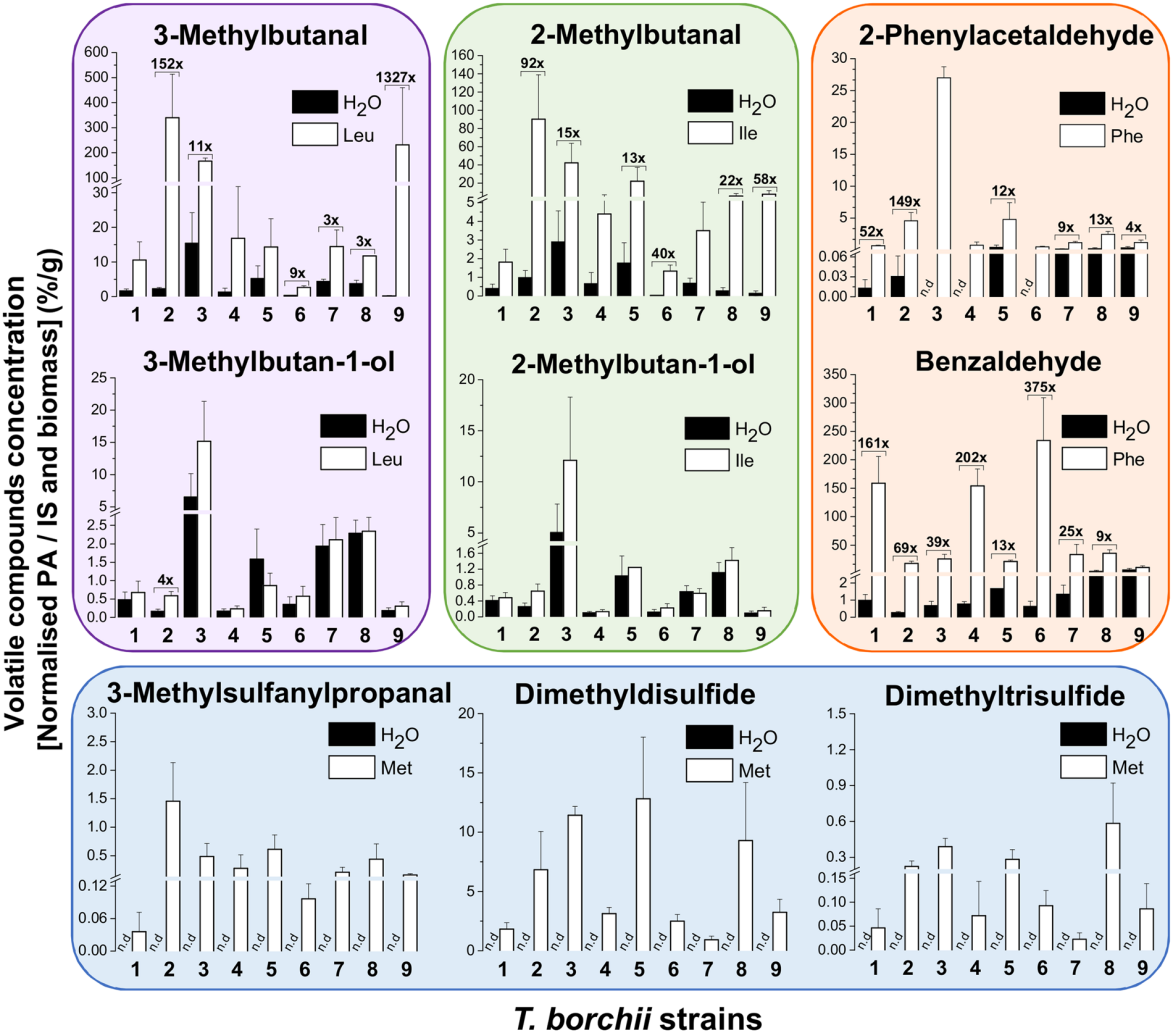
Induction of volatiles compounds derived from the Ehrlich pathway in *T*. *borchii*. Bars represent the normalised concentrations (average ± standard error for four biological replicates) of volatile compounds in nine strains of *T*. *borchii* – either unsupplemented (H_2_O) or supplemented with 20 mM amino acids namely leucine (violet), isoleucine (green), phenylalanine (orange), and methionine (blue). Concentrations of specific volatile compounds that differed between supplemented and unsupplemented strains are indicated above bars (as the “induction” x) when statistically significant (p < 0.05, Kruskal-Wallis test with α = 0.05).

Having confirmed the existence of the Ehrlich pathway in truffles, and documented an important concentration variability in resulting volatiles, we next questioned whether strains differed in their ability to produce those volatiles in the presence of amino acids. With this aim, nine mycelial strains were supplemented with non-labelled amino acids (leucine, isoleucine, phenylalanine, and methionine) and the concentration of induced volatiles was compared to the one produced by un-supplemented strains. For illustration, chromatograms of strains 5 and 7, both supplemented with Met and unsupplemented (water control) is shown in Fig. 4. Strain 5 displayed a considerably higher level of volatiles induction (DMDS, 3-methylsulfanylpropanal and DMTS) compared to strain 7. Quantifying volatiles in all samples highlighted that supplementing amino acids induced the production of volatiles to different extents depending on the strain. In Fig. 5, statistical differences in the concentrations of nine volatiles among supplemented and unsupplemented samples are indicated above bars when significant (i.e. 3-methylbutanal was 152 times higher in strain 2 supplemented with leucine compared to the unsupplemented H_2_O sample). Comparing relative concentrations among samples that emitted the highest or lowest concentration of a specific volatile compound similarly highlights statistical differences (i.e. 3-methylsulfanylpropanal was induced 41 times: strain 2_MAX_ = 1.46 ± 0.67, strain 1_MIN_ = 0.04 ± 0.03, p = 0.02, Kruskal-Wallis test with α = 0.05); 2-phenylacetaldehyde was induced 55 times: strain 3_MAX_ = 26.99 ± 1.73, strain 6_MIN_ = 0.48 ± 0.09, p = 0.02, Kruskal-Wallis test with α = 0.05). Altogether, a high variability in the production of volatiles compounds derived from the Ehrlich pathway was observed among strains upon amino acid addition, resulting in inductions of up to 1327 times compared to control samples (3-methylbutanal, strain 9).

### Sensory tests with *T*. *borchii* mycelial strains

Considering the differences in aroma profile exemplified among strains of *T*. *borchii* in our earlier experiments, we questioned if these differences would impact human-sensed aroma perception. Indeed, to have a possible impact on the overall aroma, a volatile compound needs to be detectible by the human nose (some volatile compounds are odourless) and be present above its detection threshold. A total of three sensory tests were performed to address various questions. The first test was carried out to investigate the ability of the human nose to perceive induced volatiles in *T*. *borchii* strains upon amino acid supplementation. A second test aimed at exploring the capability of the human nose to differentiate between aromas induced by a single amino acid or a mixture using a single strain. In the third test, the ability of the human nose to distinguish among strains supplemented with the same amino acid was examined. All tests were performed by 14 panellists and consisted of a triangle test (discrimination test) and an assessment of aroma attributes as described in the material and methods section.

### Induced sulfur containing volatiles are perceived by the human nose

In the first test, mycelial strain 2 was supplemented with methionine or water (control). The result of the triangle test illustrated that the panellists were able to distinguish between unsupplemented and methionine supplemented samples (p = 0.02 < α, one-sided binomial proportions test with α = 0.05), hence confirming that sulfur containing volatiles (hereafter “sulfur volatiles”) of the Ehrlich pathway were perceived by the human nose.

**Figure 6:**
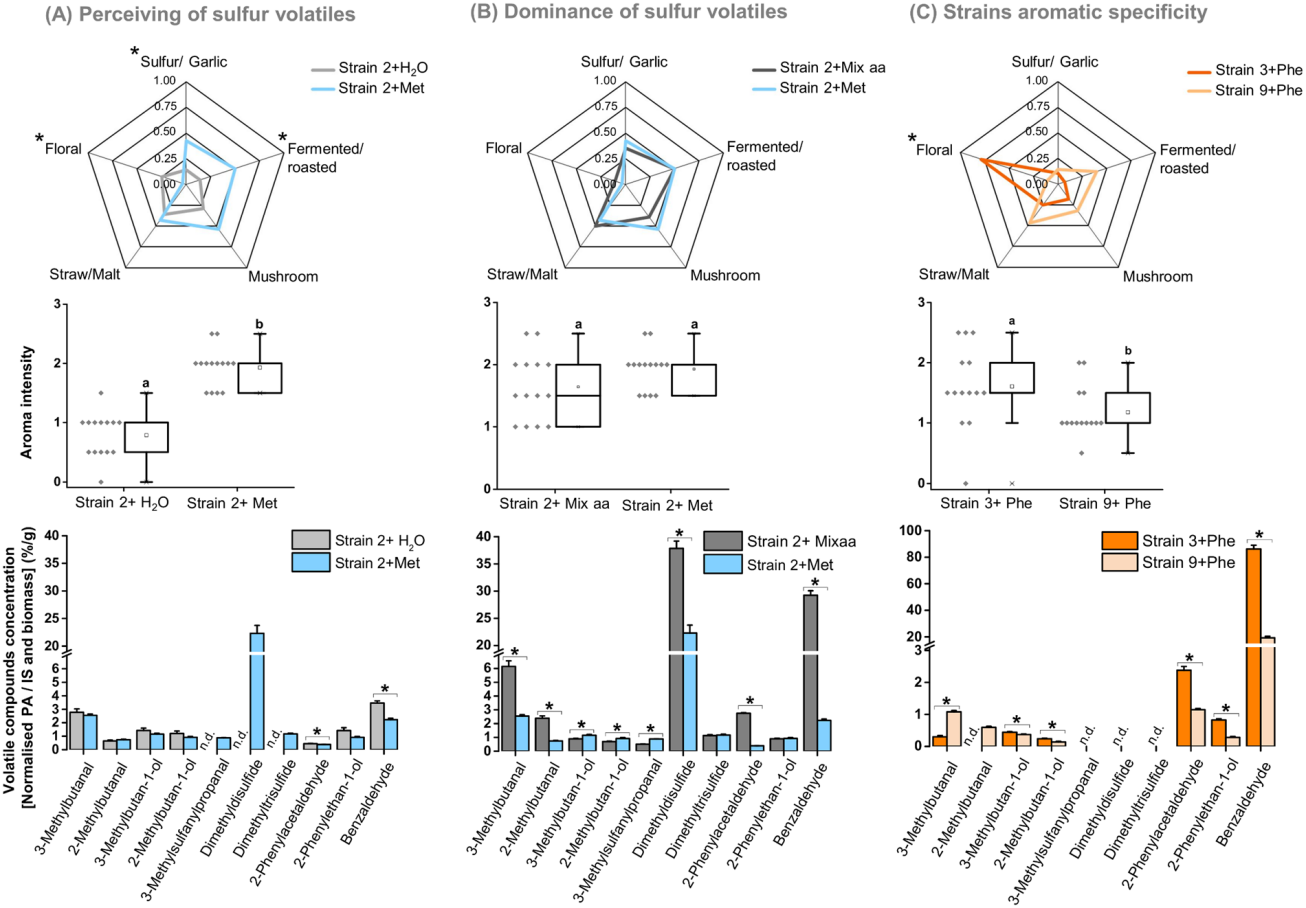
Results of sensory tests. From top to bottom, graphs illustrate aroma descriptors (spider chart), aroma intensities (box plot) and concentration of volatile compounds (bar chart), respectively. Refer to the main text for details. Statistical differences between samples (i.e. for each aroma descriptor or for aroma intensity or the concentration of specific volatile compounds) are marked with a star or with different letters (box plot) (p < 0.05, Kruskal-Wallis test with α = 0.05).

In terms of aroma attributes, differences between samples could be ascribed to sulfur and fermented/roasted notes that were highest in methionine supplemented samples and floral notes that were the highest in unsupplemented samples (Fig. 6A). Samples also differed in terms of intensity with methionine supplemented samples being the most intense (Fig. 6A). The samples were further analysed by pressure-balanced headspace-GC/MS to relate volatile profiles to the aroma impressions of the panellists (Fig. 6A). The marked sulfurous/garlicky notes reported by the panellists in the methionine supplemented sample, corresponded to the induction of sulfur volatiles, whereas the flowery notes of the unsupplemented sample corresponded to the induction of 2-phenylacetaldehyde and 2-phenylethan-1-ol.

### Induced volatiles by single or mixture of amino acids are not distinguished by the human nose

A second triangle test was performed to estimate the overall importance of sulfur volatiles compared to other (non-sulfur) aroma compounds. Essentially, the aromas induced in a single strain (strain 2 as used in the first trial) by methionine were compared to those induced by a mixture of amino acids that included methionine and also leucine, isoleucine and phenylalanine. The triangle test of the second trial illustrated that the human nose was unable to distinguish among samples supplemented with methionine only compared to those supplemented with a mixture of amino acids (that included methionine as well) (p = 0.86 > α, one-sided binomial proportions test with α = 0.05). Similarly, no significant difference in overall aroma intensity among the samples was detected (Fig. 6B). However, quantification of volatiles by pressure-balanced headspace-GC/MS highlighted the marked differences in DMDS and benzaldehyde, that were significantly higher in the sample supplemented with the amino acid mixture (Fig. 6B).

### The human nose can differentiate among strains

A third triangle test was performed to examine the ability of the human nose to distinguish between strains 3 and 9 supplemented here with phenylalanine only.

The triangle test results demonstrated that the human nose could differentiate between strains supplemented with phenylalanine (p = 0.004, one-sided binomial proportions test, α = 0.05). These findings were corroborated by the sensory test since higher floral notes were attributed to strain 3 (Fig. 6C). Profiling of volatiles revealed that the concentrations of six volatiles significantly differed among strains 3 and 9, with 2-phenylacetaldehyde, 2-phenylethan-1-ol and benzaldehyde being the highest in strain 3 (p < 0.05, Kruskal-Wallis test with α = 0.05) (Fig. 6C).

## Discussion

Genetics is a powerful tool to improve flavour and aroma of food products and/or eliminate off-flavours^23–26^. The present study demonstrated how strain selection could be used to improve truffle mycelium flavour. Type of strain however is not the only means of flavour improvement in truffles since Xiao *et al*.^20^ illustrated that desirable sulfurous, mushroom and earthy aromas could also be induced in the black truffle *T*. *melanosproum* by repeated freeze-thaw cycles.

The aroma emitted by axenic cultures of three truffle species (*T*. *borchii*, *T*. *melanosporum*, *T*. *formosanum*) have been described earlier by various groups^13,14,19,20,30,31^, yet none of those studies investigated aroma variability within the same species. Here, we used nine strains of *T*. *borchii* for the latter purpose. Of the 21 volatiles identified from *T*. *borchii* mycelia grown on malt extract (Fig. 2B), only five have been previously reported from two *T*. *borchii* strains including 3-methylbutan-1-ol, oct-1-en-3-ol, 2-phenylethan-1-ol, phenylmethanol and 2,4-bis(1,1-dimethylethyl)-phenol^13,14^. Tirillini *et al*.^14^ could nevertheless detect 28 additional volatiles including dimethyltrisulfide through dynamic headspace sampling. Similarly, ten volatiles reported in Fig. 2B have been described from axenic cultures of *T*. *melanosporum* or *T*. *formosanum*, which also produced numerous volatiles not reported here^30,31^. These similarities and discrepancies in volatile emission among our results and literature might be attributed to various factors. For example, cultural conditions might have a major impact on fungal volatiles^32,33^. In the case of axenic cultures of different truffle species (*T*. *melanosporum*, *T*. *sinense*, *T*. *indicum*, and *T*. *aestivum*), Tang *et al*.^34^ demonstrated that cultural conditions indeed had a stronger influence on the composition of emitted volatiles than species itself. Besides media composition, volatile sampling techniques might also influence volatile profiles^35^, and so does sample processing (i.e. freezing)^1,20,36^. Nevertheless, considering that in our study, all strains of *T*. *borchii* were handled in the same way, the aroma variability observed among strains (Fig. 2A,B) was without a doubt attributed to genetic differences among strains.

The influence of genetics on truffle aroma had been reported earlier in *T*. *aestivum*^21,22^ fruiting bodies for specific groups of volatiles, namely four (C_4_) and eight (C_8_) carbon-containing compounds (i.e. C_8_: oct-1-en-3-one, oct-1-en-3-ol and t-2-octenal; C_4_: butan-2-one and butan-2-ol). Our data support the influence of genetics on the 29 volatiles reported in Fig. 2B, and particularly on volatiles of the Ehrlich pathway, which could not be shown in earlier work by our group^21,22^. This apparent contradiction between our earlier results and those presented here can be explained by differences in study systems (axenic mycelial cultures versus fruiting bodies). Indeed, fruiting bodies are heavily colonized by microbes (i.e. bacteria and yeasts), which, in addition to truffles, are able to produce volatiles from the Ehrlich pathway^15^. The production of the Ehrlich-derived volatiles 2-methylbutan-1-ol, 3-methylbutan-1-ol along with some sulfur volatiles (3-methylsulfanylpropanal, dimethyl sulfide (DMS), DMDS and DMTS) have been reported from yeasts directly isolated from truffle fruiting body^37^. Besides microbes, other factors might influence fruiting body aroma (i.e. maturation^38^, geographical location^39^). Many of those factors often co-vary (i.e. genetics and geography), thus using fruiting bodies for investigating the factors behind aroma variability in truffles can only lead to limited conclusions.

The Ehrlich pathway, first described in the budding yeast *Saccharomyces cerevisiae*, consists of a three-step process, in which an amino acid is first deaminated into a α-keto acid, followed by a decarboxylation into an aldehyde and a reduction or oxidation into an alcohol or acid^17^. We demonstrated here the existence of this pathway in *T*. *borchii* using amino acids with ^13^C labelled carbons, yet, proving the origin of non-carbon atoms (i.e. sulfur, oxygen) was beyond the scope of our study. According to the “conventional Ehrlich pathway”, phenylalanine leads to two volatiles, 2-phenylacetaldehyde and 2-phenylethan-1-ol^17^. In our data (Fig. 3), the incorporation of ^13^C labels into a third volatile (benzaldehyde) hints at possible pathway differences between *Saccharomyces* yeasts and truffles. Considering that benzaldehyde might be produced from phenylalanine through both enzymatic and chemical reactions in some bacteria^40^, suggests that this might similarly occur in truffles. Identifying the exact pathway in truffles shall be the focus of further studies.

Methionine catabolism leads to the formation of sulfur volatiles through two hypothetical pathways for which candidate enzymes have been identified in the genome of *T*. *melanosporum*^10,16^. Indeed, in the “standard” Ehrlich pathway, methionine might be converted into 4-methylthio-2-oxobutyric acid (KMBA) via transamination. Subsequently, KMBA decarboxylation might lead to 3-methylsulfanylpropanal which might further non-enzymatically decompose to methanethiol (MTL). The latter might also be produced from KMBA through both chemical and enzymatic reactions by C-S lyase enzymes (i.e. cystathionine β- and γ-lyases). In the alternative “non-standard” Ehrlich pathway, methionine might directly be converted into MTL by enzymatic reactions (i.e. methionine γ-lyase). In both standard and non-standard pathways, MTL can spontaneously oxidize to DMS, DMDS and DMTS^10^. The induction of 3-methylsulfanylpropanal observed in our data (Fig. 3) supports the existence of at least the standard Ehrlich pathway for methionine catabolism in *T*. *borchii*. By contrast, the absence of DMS, a typical sulfur volatile of many truffle species^10^ from our mycelial cultures might be interpreted in two ways. On one side, DMS might not be induced by the cultural conditions used here, and different conditions might have favoured it as reported for *T*. *melanosporum* and *T*. *formosanum*^30,31^. On the other side, DMS in truffle fruiting bodies might actually be exclusively produced by bacteria (and not by the truffle mycelium), as demonstrated for some cyclical sulfur volatiles in *T*. *borc*hii^12^. Either hypothesis will require a more detailed characterization of methionine catabolism pathway to be tested.

Volatiles produced through the Ehrlich pathway are well-known food odorants^6^. For example, taken as pure compounds, 2-phenylethan-1-ol has a rose-like odour, 3-methylbutan-1-ol is reminiscent of cheese and whiskey and sulfur volatiles (i.e. DMS, DMDS, DMTS and 3-methylsulfanylpropanal) smell like garlic, rotten food, and cooked potatoes^8,15^. Aroma perception of specific food depends on the interplay among odorants and on their respective concentrations^6^, yet, typically, only a small fraction of all volatiles contribute to human-perceived aroma. Our sensory tests illustrated the importance of methionine in the formation of sulfur containing odorants in axenic truffle cultures. Indeed, sulfur volatiles are also major odorants in truffle fruiting bodies^7,8,15^. In our sensory tests, panellists predominantly used sulfur/garlicky and fermented/roasted notes to describe the aroma of mycelium supplemented with methionine. Even if not determined here, this strongly suggests that the concentration of sulfur volatiles were higher than their detection threshold (odour activity value (OAV) > 1) in methionine supplemented samples. This is further supported by the fact that sulfur volatiles tend to have much lower odour thresholds compared to other volatiles of the Ehrlich pathway (i.e. odour threshold in water (part per billion) of sulfur volatiles DMDS (0.16–12)^41–43^ and DMTS (0.005–0.01)^41,43^ versus non-sulfur volatiles such as 2-phenylethan-1-ol (750–1100)^44,45^ and 3-methyl-1-butanol (250–300))^44,45^.

Panellists could further differentiate among mycelial strains supplemented with the same amino acids. This illustrates that genetics does not only influence volatile production in truffles as highlighted earlier, but that those differences have an impact on the overall aroma characteristics. Similar findings of strains influence on aroma characteristics have been also demonstrated for strawberries and baker’s yeast^46,47^, among numerous other examples. Specifically, in domesticated strawberries, an insertional mutation was shown to result in flavour loss compared to wild strawberries^46^. Similarly, some non-conventional yeast strains markedly vary in aroma profile (i.e. concentrations of 2-phenylacetaldehyde and 2-phenylethan-1-ol) compared to the *S*. *cerevisiae* strain traditionally used in bakery^47^.

Our findings illustrate how strain selection might be used to identify truffle strains with more desirable aroma and flavour. This is specially promising since *T*. *borchii* is the first species that has been successfully used to mycorrhize seedlings with axenic mycelial cultures instead of ascospores^48^. Overall, our results might also lead to the production of better quality truffle flavour and a higher consumer acceptance of truffle-flavoured food products (i.e. truffle-flavoured olive oil) that currently predominantly contain synthetic flavours.

## Materials and Methods

### Biological material

A total of 9 strains (axenic cultures) of *T*. *borchii* and one fruiting body (FB) were used in this study as described in Table 1.

For preparation of liquid cultures, *T*. *borchii* mycelia were pre-grown on malt extract (ME) agar (pH 7.0) [formula used per liter: malt extract 10 g (Difco & Becton Dickinson, Heidelber, Germany) and agar 15 g (Carl Roth, Karlsruhe, Germany)] for two months as described by Splivallo *et al*.^21^, and half a colony transferred to 100 ml Erlenmeyer flasks containing 30 ml of ME broth (1%, pH 7.0). Liquid cultures were homogenized with a sterile grinder at 17,000 rpm (T 18 digital ULTRA-TURRAX, IKA, Staufen, Germany) for 10 s, and incubated at 23 °C for two months. Sample handling for subsequent experiments is illustrated in Fig. 1. along with the number of replicates used for each experiment.

### Comparing the volatile profiles of different *T*. *borchii* mycelial cultures

Two-month-old mycelial cultures and malt extract broth without mycelium (negative controls) were homogenized at 17,000 rpm as described above, an aliquot of 5 ml in a 50 ml tube was subsequently pelleted by centrifugation (12,000 g for 10 min at 4 °C). The supernatant was discarded and a biomass of 200 ± 0.002 mg (wet weight) was transferred to a 20 ml SPME vial sealed with a screw cap and a silicon/polytetrafluoroethylene (PTFE) septum (VWR, Germany). Medium control samples were prepared by transferring 100 µl of malt extract to SPME vials. One fruiting body sample (originally frozen, but allowed to equilibrate to room temperature for 2 h before analysis) was also included for volatile analysis (300 ± 0.002 mg per SPME vial).

Volatiles extraction was performed by SPME in an autosampler (PAL RSI 85, CTC Analytics AG, Switzerland). Samples were first preheated to 60 °C for 20 min prior to extraction. Extraction was then done with an SPME fibre (PDMS/DVB/CARB Agilent Technologies, Waldbronn, Germany) exposed for 15 min in the headspace of the sample. Empty SPME vials were regularly inserted between samples to make sure no carry-over occurred. Volatiles were profiled by GC/MS (Agilent 7890B GC system equipped with a 5977B quadrupole MS detector (Agilent Technologies, Waldbronn, Germany). The SPME fibre was desorbed in the GC inlet (250 °C) in splitless mode and volatiles were separated on a capillary column (HP-5MS Agilent 19091S-433UI 0.25 mm × 30 m × 0.25 μm) by using the following program: start at 40 °C and hold for 5 min, ramp at a rate of 3 °C min^−1^ to 160 °C, ramp at a rate of 50 °C min^−1^ to 260 °C, hold for 1 min (total run time: 53 min). The carrier gas was helium with a flow rate of 1.2 ml min^−1^. MS parameters were adjusted to a mass-to-charge ratio (m/z) scan range from 50 to 350, in an etune mode, ion M^+^, with electron energy of 70 eV (MS source 230 °C, MS quad 150 °C).

Chromatograms were visualized with the Agilent Mass Hunter Qualitative Analysis software (version: B.07.00). To eliminate the shift in retention time due to the machine drifts, peaks were aligned using Tagfinder software version 4.1^28^. The intensity threshold was set to 3,000 but other parameters were the same as previously described in Sherif *et al*.^49^. As an output, Tagfinder created a matrix with sample names in columns, and TAGs (specific m/z values within a specific retention time window) in rows. Background noise (three times the values of the medium control samples (ME without mycelium)), was subtracted from all samples and TAGs for each sample were normalized by dividing their intensity by the total ion current (TIC) (Supplementary Table S1). Based on Supplementary Table S1, principal component analysis (PCA) was produced using the Past software version 3.04^50^. To create the heat map in Fig. 2B from Supplementary Table S1, a non-parametric test in R (version 3.2.3)^29^ was used to identify TAGs that significantly differed among samples (p < 0.05, Kruskal-Wallis with α = 0.05). Moreover, to avoid a biased representation in the heatmap towards tags with the highest intensities, TAG in single rows were divided by their maximum TAG intensity in that row. This resulted in relative concentrations between 0 and 1.

### Identification of volatile compounds

Volatiles were identified by comparison of their mass fragmentation patterns with mass spectra databases (National Institute of Standards and Technology (NIST) library v. 2.0, Gaithersburg, USA), and by comparison of Kovats retention indices (calculated from n-alkanes) to literature values (http://www.pherobase.com/database/kovats/kovats-index.php, http://webbook.nist.gov/chemistry/gc-ri/). Moreover, authentic standards were used to confirm the identity of the following volatiles by GC/MS: 2-methylpropan-1-ol, 2-methylbutan-1-ol, 3-methylbutan-1-ol, oct-1-en-3-ol, 2-methylbutanal, 3-methylbutanal, 2-phenylethan-1-ol, benzaldehyde, 2-phenylacetaldehyde, DMDS, DMTS.

### Confirming the Existence of the Ehrlich Pathway in *T*. *borchii* with ^13^C labelled amino acids

Supplementation experiments with ^13^C isotope labelled amino acids were performed with three mycelial strains (2, 3 and 5 in Table 1). Mycelial strains pre-grown in 30 ml malt extract liquid cultures were homogenised with a grinder and washed and pelleted three times with 5 ml minimal medium. Minimal medium contained the following components (concentrations are given in mg/l): MgSO_4_.7H_2_O: 731, KNO_3_: 80, KCl: 65, KH_2_PO_4_: 5, MnCl_2_.4H_2_O: 6, ZnSO_4_.7H_2_O: 4.52, H_3_BO_3_: 1.5, CUSO_4_. 5H_2_O: 0.26, (NH_4_)_6_Mo_7_O_24_.4H_2_O: 0.0046, Ca (NO_3_)_2_.4H_2_O: 288, NaFeEDTA: 8, glycine: 3, thiamine: 0.1, pyrodoxine: 0.1, nicotinic acid: 0.5, myo-inositol: 50, KI: 0.75, sucrose: 10,000. After washing, mycelial pellets were dissolved in 5 ml minimal medium and 1.5 ml of this culture was transferred to an SPME vial. Vials were supplemented with 0.5 ml of an aqueous solution (sterile filtered) containing ^13^C labelled L-methionine, L-leucine, L-isoleucine and L-phenylalanine (labels are shown in Fig. 3, final concentration of 5 mM in SPME vials) (Cambridge isotope laboratories, Andover, USA). Samples were incubated at room temperature on a 100 rpm orbital shaker (Neolab, Heidelberg, Germany) for 24 h in the dark. After incubation, mycelial volatiles were profiled by pressure-balanced headspace-GC/MS as described hereafter.

Volatiles were profiled using a Clarus 680 GC coupled to quadrupole Clarus SQ 8 C MS detector (PerkinElmer, MA, USA) equipped with a pressure-balanced headspace- autosampler (Headspace Sampler TurboMatrix 40, Perkin Elmer, MA, USA). Volatiles were extracted by pressure-balanced headspace in trap mode using the following program: 20 ml SPME vials sealed with a septum were pre-heated for 20 min at 70 °C (to increase the volatility of the analytes), then the vials were pressurized with helium (to 105 kPa for 2 min). 4-Bromofluorobenzene (100 µl of a 27.2 ppm IS in N2 (mol/mol)) was used as an internal standard (IS). The IS gas was injected into the vials prior to volatile adsorption by the trap (TurboMatrix air monitoring trap, Perkin Elmer) (loop load: 0.5 min, loop equilibration: 0.4 min, inject time 0.5 min). Volatiles were thermally desorbed from the trap and injected in the GC by increasing the temperature from 30 °C to 280 °C. Separation was performed on a capillary column Elite-5MS (30 m × 0.25 mm i.d., 1.00 μm film thickness, Perkin Elmer). The GC oven temperature program was: start at 50 °C and hold for 1 min, 15 °C min^−1^ to 180 °C and hold for 1 min, 100 °C min^−1^ to 300 °C and hold for 5 min (total run time: 16.87 min). The carrier gas was helium with constant pressure at 75 kPa. MS operated electron impact ionisation (EI) conditions (70 eV) with scan range from m/z 50-300 (MS source 200 °C). Turbo Mass software (version: 6.1.0, PerkinElmer) was used to visualize the chromatograms and the mass spectrums. Mass spectra of mycelial strains supplemented with ^13^C labelled amino acids was compared to the mass spectra of non-labelled amino acids supplemented mycelia to inspect whether the labels were integrated into the targeted volatiles (see Supplementary Fig. S1).

### Testing the induction of Ehrlich volatiles in *T*. *borchii*

The supplementation experiment with non-labelled amino acids was carried out using all the nine mycelial strains of Table 1. Mycelial cultures and controls with no mycelium in ME broth were homogenized with a sterile grinder and were divided into aliquots of 2 ml in SPME vials. Vials were supplemented with 0.4 ml of an aqueous solution containing non-labelled methionine, leucine, isoleucine and phenylalanine (sterile filtered, with a final concentration of 20 mM in SPME vials) (Carl Roth, Karlsruhe, Germany) or water (control). Samples were kept at room temperature on a 100 rpm orbital shaker for 24 h in the dark. Mycelial volatiles were profiled as for the samples containing labelled amino acids. To determine the mycelial biomass, 3 ml of each homogenized culture was transferred to 50 ml Falcon tube, the mycelium was separated by centrifugation from its supernatant and wet weight was recorded. Peak areas (PA) of target volatiles were subsequently normalized to the peak area of the internal standard and to the biomass of each sample (Fig. 5).

### Sensory tests with *T*. *borchii* mycelial strains

Three sensory tests were performed in total, aimed at various objectives. A first test was done to evaluate if the human nose perceived induced volatiles in *T*. *borchii* strains supplemented with methionine (comparison: strain 2 either unsupplemented (water control)) or supplemented with 5 mM methionine. The second test was performed to investigate whether the human nose could differentiate between the aromas induced by methionine (strain 2, 5 mM) or a mixture of amino acids (strain 2 and leucine, isoleucine, phenylalanine, and methionine, each 5 mM) in the same strain. A third test assessed the ability of the human nose to distinguish between strains (strains 3 and 9, both supplemented with 5 mM phenylalanine).

For all the sensory tests, panellist (14 individuals, 6 women and 8 men) were presented with a questionnaire comprising two parts: a discriminatory test (“triangle test” as described in O’Mahony *et al*.^51^) and assessment of aroma attributes. For the triangle test, each panellist was presented with three samples (two identical and one “different”) and asked to identify the two samples judged the most similar by their smells. Results of the triangle test were analysed using a one-sided binomial proportions test^52^. For the assessment of aroma attributes, each panellist was presented with four samples (two replicates of each sample). They were asked to indicate the presence of the following aroma attributes; sulfur/garlic, mushroom, floral, straw/malt, fermented/roasted, followed by rating the “overall aroma intensity” using a four-point category scale (0 indicating ‘no smell’ and 3 ‘very intense smell’). These aroma attributes were provided to the assessors as representative aroma attributes typically used to describe the volatiles of Fig. 5. For each aroma descriptor, frequencies were expressed as a percentage of total possible counts (14 panellist × 2 replicates = 28 counts), yielding values between zero and one. Volatile profiles from the samples used for sensory analysis were generated by pressure-balanced headspace -GC/MS as previously described.

### Ethics statement

Use of human subjects for this study was reviewed by the Ethics Committee of the medical department of the University of Frankfurt and was granted exempt status. Informed consent was obtained from all participants.

### Data Availability

The data generated or analysed during this study are included in this published article (and its Supplementary Information files).

## Electronic supplementary material

Table S1

Supplementary Figure S1
