# Supplementary material for: Improving truffle mycelium flavour through strain selection targeting volatiles of the Ehrlich pathway
Source: Sci Rep. 2018 Jun 18;8:9304. doi: 10.1038/s41598-018-27620-w (PMC6006436; doi:10.1038/s41598-018-27620-w)
Supplement: Supplementary file 2 — Supplementary Figure S1 [file 41598_2018_27620_MOESM2_ESM.pdf]

Improving truffle mycelium flavour through strain selection targeting volatiles of the Ehrlich pathway

Maryam Vahdatzadeh[1], Richard Splivallo[1,*]

[1] Goethe University Frankfurt, Institute for Molecular Biosciences, 60438 Frankfurt, Germany

* Corresponding author: richard.splivallo@a3.epfl.ch

Fig. S1 – Part 1

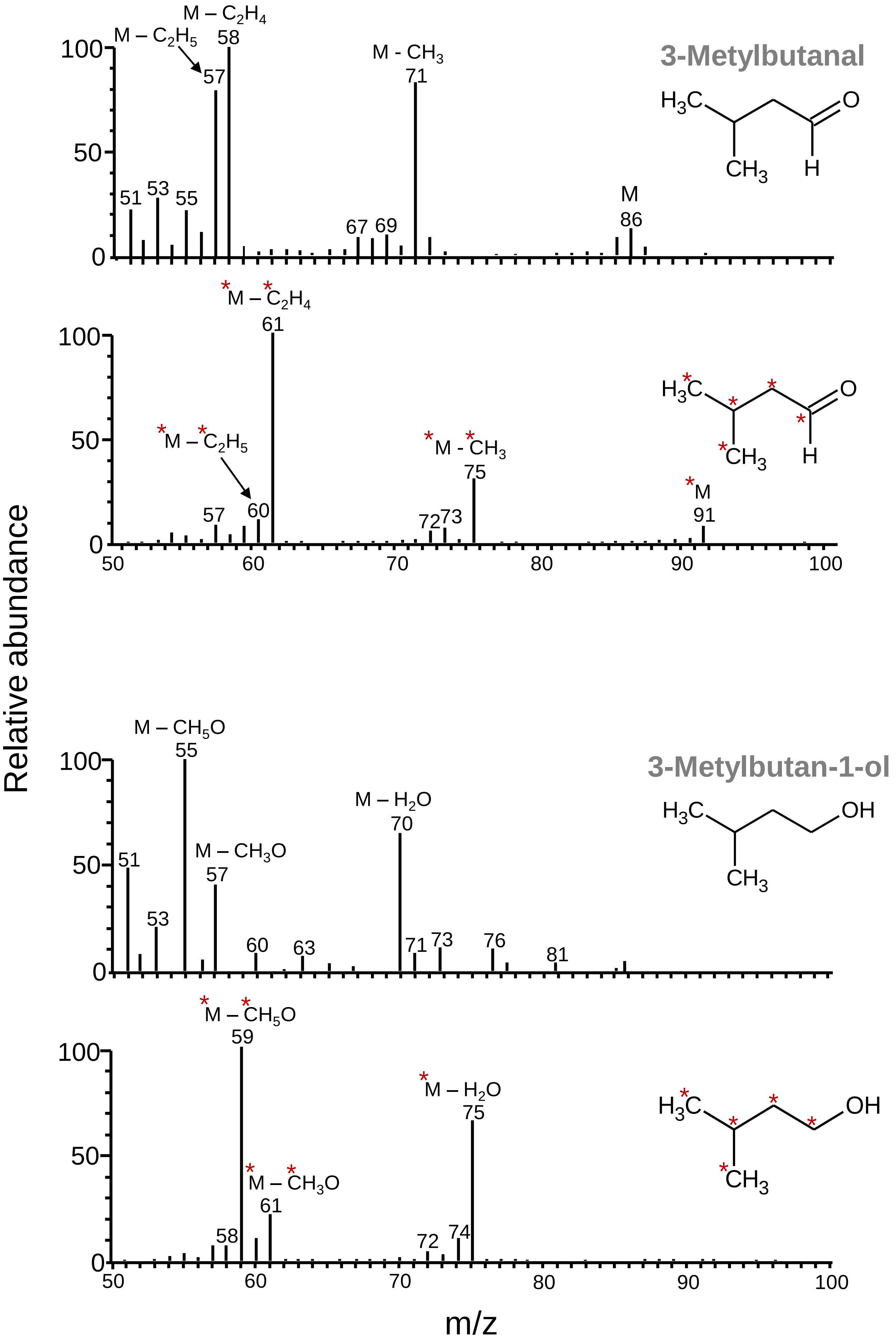

3-Metylbutanal
3-Metylbutan-1-ol
Relative abundance
m/z
M – C2H5
M – C2H4
M - CH3
M
M – CH5O
M – CH3O
M – H2O

Fig. S1 – Part 2

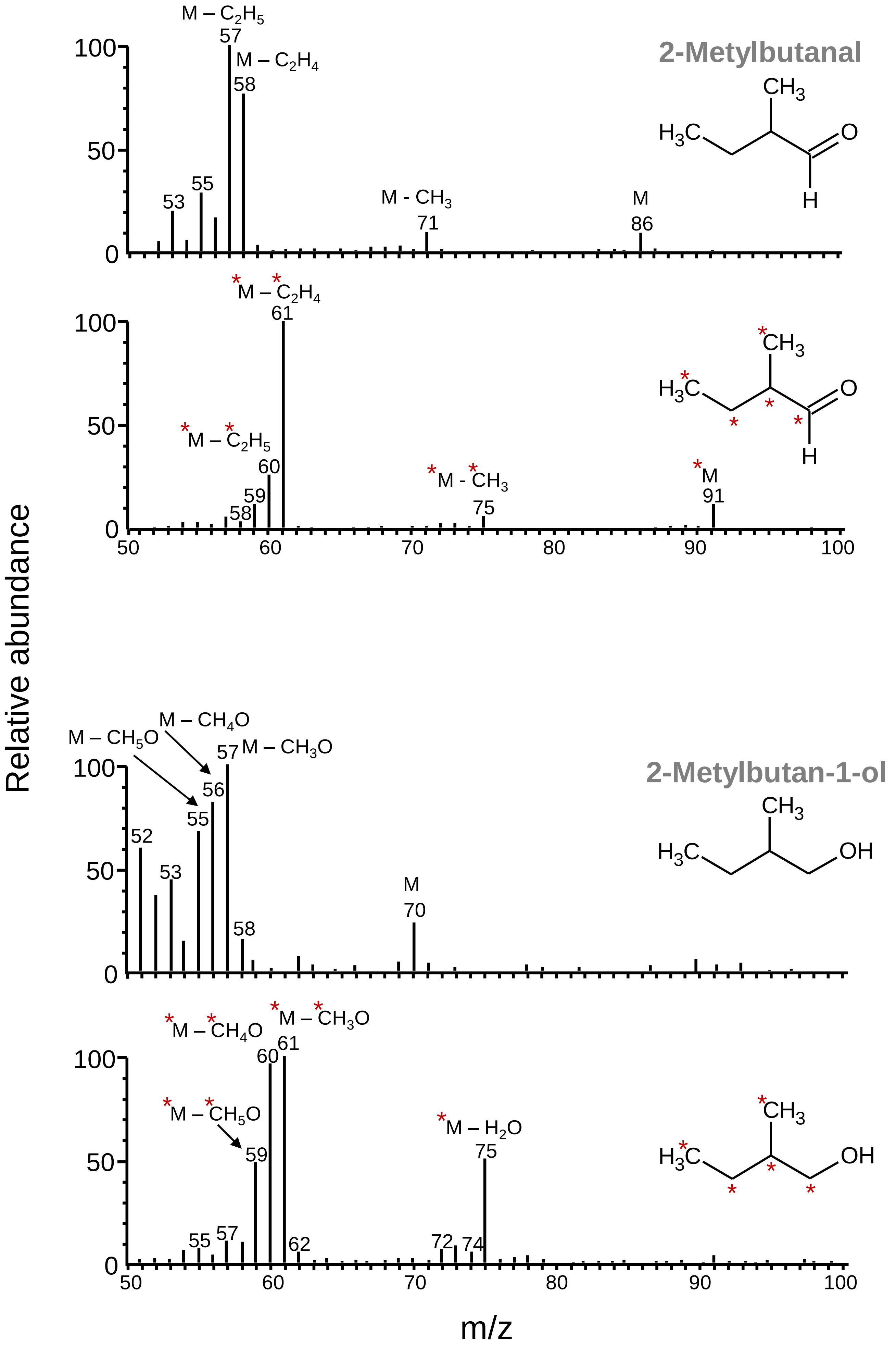

2-Metylbutanal
M – C2H5
57
M – C2H4
58
53
55
M - CH3
71
M
86
CH3
H3C
O
H
*M – *C2H4
61
*M – *C2H5
60
59
58
*M - *CH3
75
*M
91
2-Metylbutan-1-ol
M – CH4O
M – CH5O
57
M – CH3O
56
55
52
53
58
M
70
OH
*M – *CH4O
*M – *CH3O
61
60
*M – *CH5O
59
*M – H2O
75
55
57
62
72
74
Relative abundance
m/z
0
50
100
60
70
80
90

Fig. S1 – Part 3

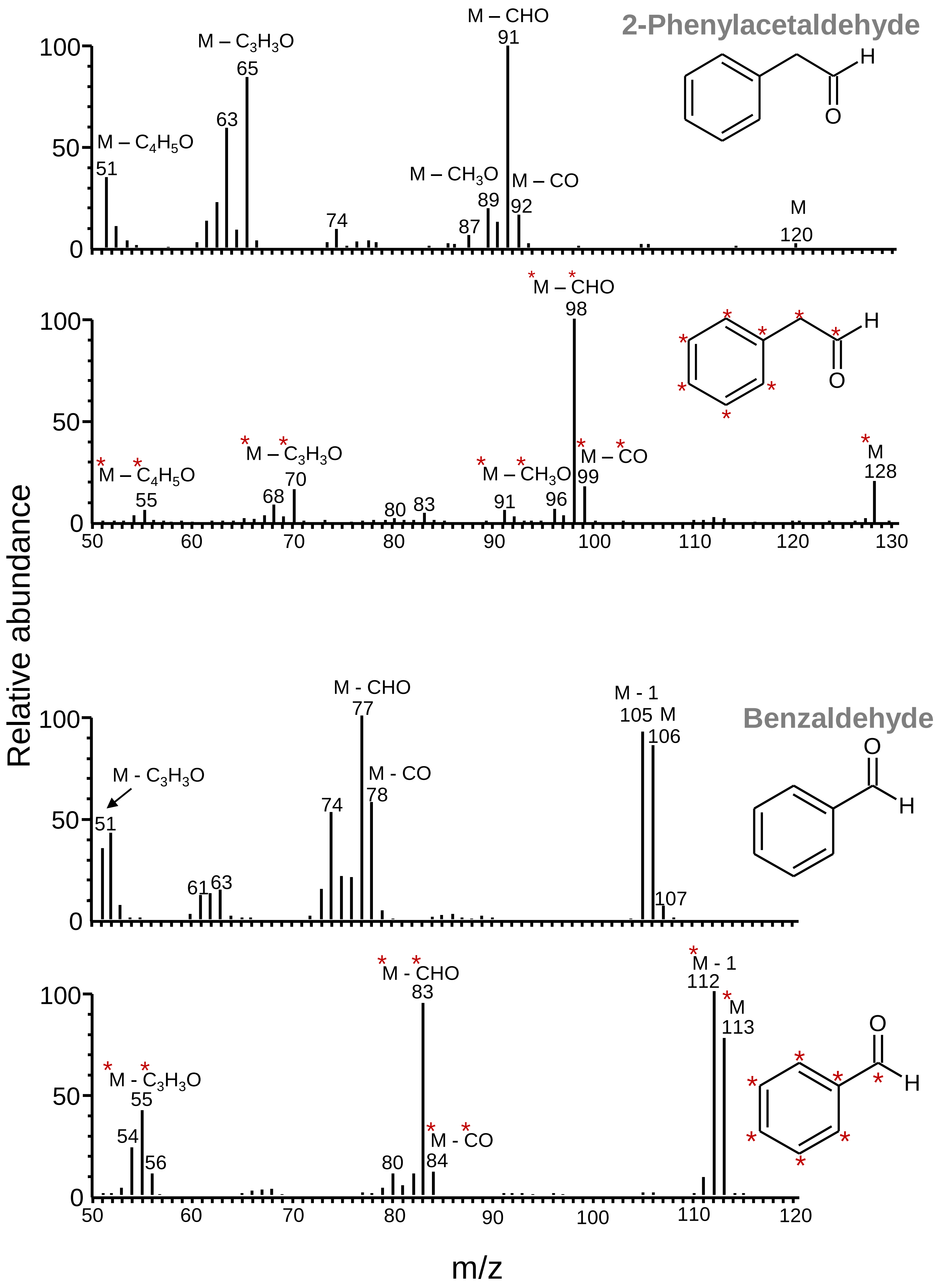
2-Phenylacetaldehyde
M – CHO
91
M – C_3H_3O
65
63
M – C_4H_5O
51
74
M – CH_3O
89
87
M – CO
92
M
120
*M – *CHO
98
*M – *C_4H_5O
55
*M – *C_3H_3O
70
68
80
83
*M – *CH_3O
91
96
*M – *CO
99
*M
128
Benzaldehyde
M - CHO
77
M - 1
105
M
106
M - C_3H_3O
51
M - CO
78
74
61
63
107
*M - *CHO
83
*M - 1
112
*M
113
*M - *C_3H_3O
55
54
56
*M - *CO
84
80
O
H
Relative abundance
m/z

Fig. S1 – Part 4

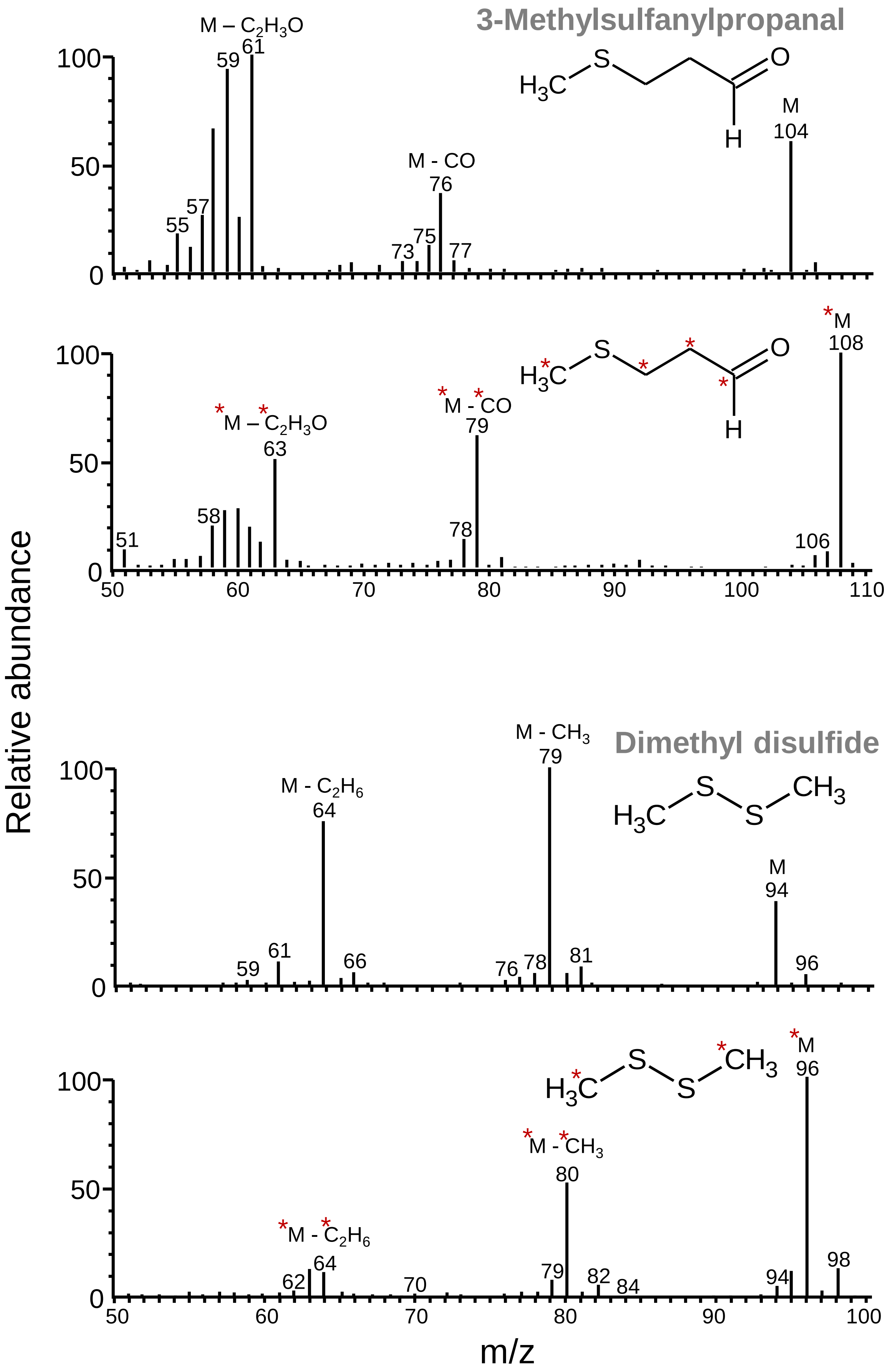
3-Methylsulfanylpropanal
Dimethyl disulfide
Relative abundance
m/z
M – C_2H_3O
M - CO
M
*M – *C_2H_3O
*M - *CO
*M
M - CH_3
M - C_2H_6
*M - *CH_3
*M - *C_2H_6

Fig. S1 – Part 5

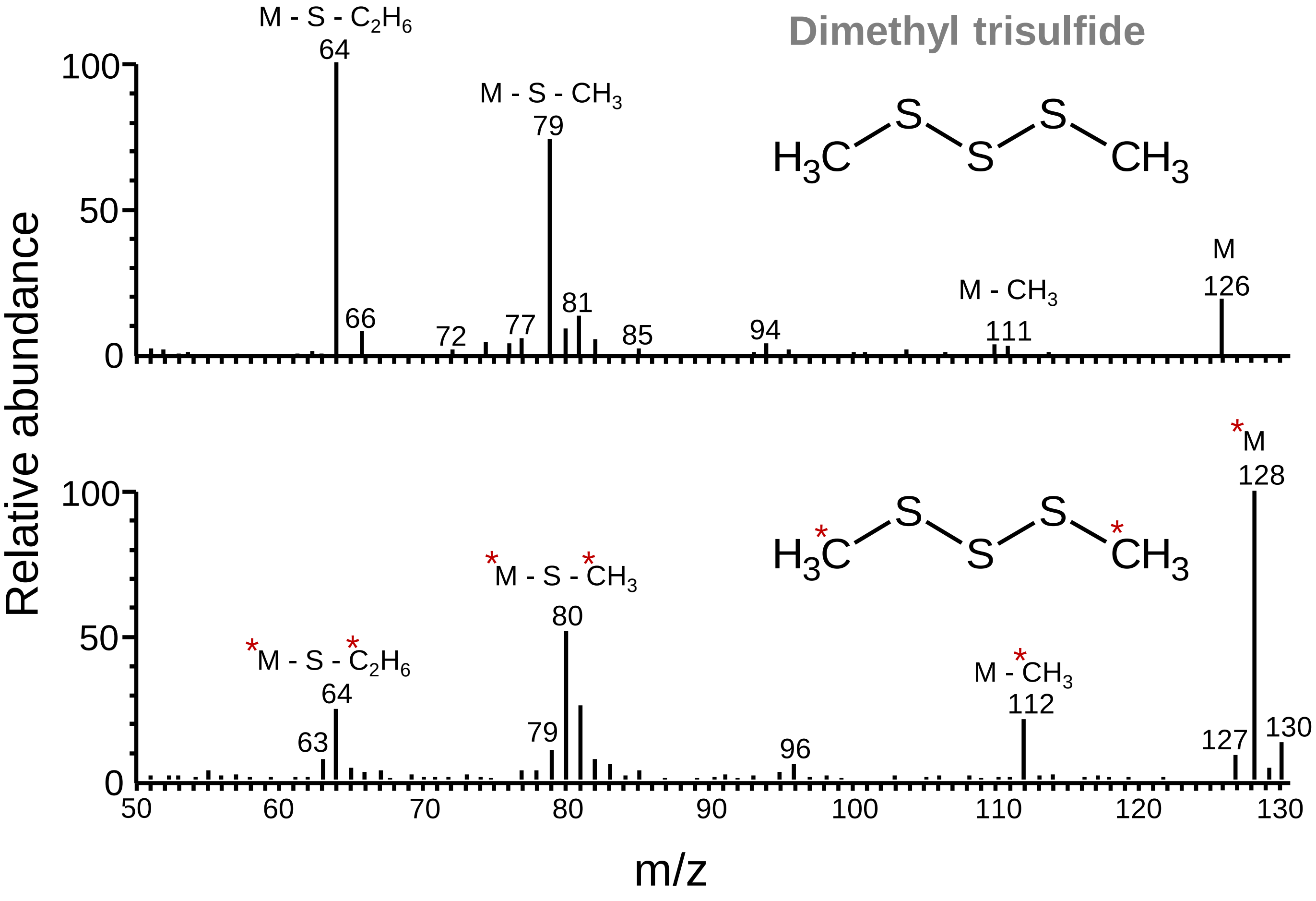

Figure S1. Demonstrating the existence of the Ehrlich pathway in *T. borchii*.

Mass spectra of volatile compounds derived from the Ehrlich pathway upon supplementing mycelial cultures with unlabelled and ^{13}C labelled (*) amino acids (leucine, isoleucine, phenylalanine, and methionine at 5 mM). Mass spectra illustrate the full incorporation of the labelled carbon atoms into target volatile compounds. Results for strains 2, 3 and 5 were equivalent.
